# Kidney function and prescribed dose in middle‐aged and older patients starting selective serotonin reuptake inhibitors

**DOI:** 10.1002/pds.5515

**Published:** 2022-08-10

**Authors:** Nanbo Zhu, Alexander Lisinski, Tyra Lagerberg, Kristina Johnell, Hong Xu, Juan Jesús Carrero, Zheng Chang

**Affiliations:** ^1^ Department of Medical Epidemiology and Biostatistics Karolinska Institutet Stockholm Sweden; ^2^ Department of Pharmacology, Institute of Neuroscience and Physiology, Sahlgrenska Academy University of Gothenburg Gothenburg Sweden; ^3^ Division of Clinical Geriatrics, Department of Neurobiology Care Sciences and Society, Karolinska Institutet Stockholm Sweden

**Keywords:** chronic kidney disease, dose adjustment, estimated glomerular filtration rate, prescribed dose, selective serotonin reuptake inhibitors

## Abstract

**Purpose:**

To avoid adverse drug reactions, dose reductions are recommended when prescribing selective serotonin reuptake inhibitors (SSRIs) to patients with impaired kidney function. The extent of this practice in routine clinical care is however unknown. We aimed to evaluate the starting and maintenance SSRI doses prescribed to patients stratified by levels of kidney function in real‐world practice.

**Methods:**

Using data from the Stockholm CREAtinine Measurements (SCREAM) project, we identified 101 409 new users of antidepressants (including 52 286 SSRI users) in the region of Stockholm during 2006–2019, who were ≥50 years of age and had a recent creatinine test taken in order to estimate glomerular filtration rate (eGFR). SSRI dose reduction was defined as a prescribed SSRI dose of ≤0.5 defined daily doses, according to current recommendations. We examined the associations between eGFR and reductions in initial dose and maintenance dose of SSRIs using logistic regression models.

**Results:**

Overall, reductions in initial and maintenance dose were observed among 54.1% and 34.1% of new SSRI users. Nevertheless, about 40% of individuals with an eGFR <30 ml/min/1.73 m^2^ were prescribed an SSRI without dose reduction. After adjusting for age and other covariates, lower eGFR was associated with moderately higher odds of dose reduction, for both initial and maintenance dose. Compared to individuals with an eGFR of 90–104 ml/min/1.73 m^2^, the adjusted odds ratios for those with an eGFR <30 ml/min/1.73 m^2^ were 1.18 (95% CI: 1.03, 1.36) for initial dose reduction, and 1.49 (1.29, 1.72) for maintenance dose reduction. Stratified analyses showed stronger associations between lower eGFR and SSRI dose reduction among individuals aged 50–64 years and in those receiving prescriptions from psychiatric care.

**Conclusions:**

Lower kidney function was moderately associated with a reduced SSRI dose, independently of age. Prescribing SSRIs to middle‐aged and older patients should not only consider patients' age but also their kidney function.

**Key Points**

- Dose reductions are recommended when prescribing SSRIs to patients with impaired kidney function, but to what extent these recommendations are followed in routine clinical care is unknown.
- In this population‐based cohort study, lower kidney function was moderately associated with reduction in SSRI dosing. Still, two in five patients with moderate to severe loss of kidney function received an SSRI prescription without a reduced dose.
- Our results highlight a gap between recommendations and real‐world prescribing practices for those with impaired kidney function.

**Plain Language Summary**

When prescribing selective serotonin reuptake inhibitors (SSRIs) to patients with impaired kidney function, dose reduction is recommended in order to avoid adverse drug reactions. In this study, we assessed to what degree these recommendations are followed in routine clinical care. The study found that patients with lower kidney function were more likely to be prescribed a reduced SSRI dose. However, still two fifths of patients with moderate to severe loss of kidney function received an SSRI prescription without dose reduction. The findings of this study highlight a gap between SSRI dose reduction recommendation and real‐world prescribing practice.

## 1 INTRODUCTION

Chronic kidney disease (CKD) is a highly prevalent condition affecting an estimated 10% of the global population.^1^ Defined as persistent abnormalities of kidney structure or function,^2^ CKD becomes more common with increasing age, and it is often comorbid with other medical conditions.^3^ Depression is one of the most common mental disorders, which is reported to be present in approximately a quarter of patients with CKD,^4^ a proportion substantially higher than in the general population. Accumulating evidence suggests that comorbid depression is associated with poor prognosis in patients with CKD, including progression to dialysis, hospitalization, and death.^5^, ^6^, ^7^

Antidepressant drugs are the main pharmacological treatment for depression, with selective serotonin reuptake inhibitors (SSRIs) as the most frequently prescribed type in most countries. Treatment guidelines for depression have highlighted the necessity to identify and address the potential interplay between depression and co‐occurring medical conditions.^8^, ^9^, ^10^, ^11^ Of note, antidepressant use in patients with CKD could have a different risk–benefit profile than in the general population, due to altered drug clearance, prolonged half‐life, and increased risk of drug–drug interactions.^12^, ^13^ Although they typically undergo hepatic metabolism, many antidepressants have active metabolites that are renally excreted and may thus accumulate in patients with CKD.^14^ In fact, drug clearance for several antidepressants including bupropion, reboxetine, and venlafaxine, is markedly reduced in patients with CKD.^15^

Concerns have been raised about adverse drug reactions of antidepressants in patients with impaired kidney function, particularly among elderly adults.^16^, ^17^ Clinical guidelines and expert opinions suggest that dose reduction is required for SSRI treatment in patients with CKD.^13^, ^15^, ^18^, ^19^, ^20^, ^21^ For instance, the European Renal Best Practice guideline recommends reducing the dose of sertraline by half for patients with CKD stage 5.^15^ Other prescribing guidelines suggest starting at a low dose for escitalopram in CKD stages 4–5,^20^, ^21^ and some experts advise a 50% lower initial dose of citalopram.^13^ However, it is largely unknown to what extent these recommendations are followed in routine clinical settings.^22^

Considering that kidney function declines in the aging process and it is common that drug therapy is started with a low dose in the elderly population, the present study specifically aimed to evaluate the association between kidney function and prescribed SSRI doses while accounting for age, using a general population sample with valid laboratory measures of kidney function from Stockholm, Sweden.

## 2 METHODS

### 2.1 Study population

This study used data from the Stockholm CREAtinine Measurements (SCREAM) project, a healthcare utilization cohort that includes all residents in the region of Stockholm accessing any form of healthcare during 2006–2019.^23^ Laboratory tests were linked to a variety of health registers, including the Stockholm regional healthcare data warehouse (VAL), the Swedish Prescribed Drug Register (PDR) and the Swedish Renal Register (SRR), for complete information on demographics, clinical diagnoses, healthcare utilization, prescribed drugs, and validated kidney replacement therapy endpoints. The PDR provides information on all dispensed prescriptions to the entire Swedish population since July 2005, including drug identity (classified by Anatomical Therapeutic Chemical [ATC] codes), dosage, date of dispensing, and the prescriber's profession and practice.^24^ The study used only de‐identified data, and thus, informed consent was not required. This study was approved by regional ethics review boards and adhered to the Declaration of Helsinki.

For the present study, we identified all individuals aged ≥50 years who collected an incident prescription of antidepressant drugs (ATC code: N06A) during 1 July 2006 to 31 December 2019. We chose this age threshold because abnormalities in kidney function are less common at younger ages and creatinine may also be less frequently measured in younger adults. Incident prescription was defined as dispensation of any antidepressant for the first time during the study period, provided that the individual had no previous recorded antidepressant dispensations. Since the Swedish PDR records prescriptions from July 2005, all included individuals had a period of at least 1 year free of antidepressant dispensations. The dispensation date of antidepressant prescriptions served as the index date. Exclusion criteria were missing information on prescription dosage, receiving more than one antidepressant on the index date, or lack of a serum/plasma creatinine measurement at the time of drug dispensing or during the previous 12 months.

### 2.2 Exposure

Kidney function was assessed by a single estimated glomerular filtration rate (eGFR), which was calculated using the most recent test of serum or plasma creatinine prior to the index date. Creatinine was measured using either enzymatic or corrected Jaffe method, both methods being traceable to isotope dilution mass spectroscopy standards. We excluded inpatient creatinine measurements, as well as implausible values (<25 or >1500 μmol/L). The 2009 Chronic Kidney Disease Epidemiology Collaboration creatinine equation was used to calculate eGFR,^25^ without applying the correction for race, as this is not recorded in Swedish registers. Following the Kidney Disease Improving Global Outcomes criteria and prior studies,^2^, ^26^, ^27^ levels of kidney function were categorized as: ≥105, 90–104, 60–89, 45–59, 30–44, and <30 ml/min/1.73 m^2^. The category with eGFR 90–104 ml/min/1.73 m^2^ was chosen as the reference, based on previous studies showing minimal health risks in this category.^28^

### 2.3 Outcomes

Information on the drug class, prescription dosage, and source of prescription (i.e., primary care, non‐psychiatric specialist care, or psychiatric care) was retrieved from the PDR. Antidepressants were classified as: tricyclic antidepressants (TCAs; ATC codes: N06AA); selective serotonin reuptake inhibitors (SSRIs; N06AB); serotonin–norepinephrine reuptake inhibitors (SNRIs; N06AX16 or N06AX21); and other antidepressants (other N06A drugs). Only the first antidepressant prescription from the index date was analyzed. The prescribed daily dose was first extracted as the number of pills per day, which was estimated from “free‐text” prescription information using a machine‐learning algorithm,^29^ and then transformed to the number of defined daily doses (DDDs) per day, according to the amount of substance per pill derived from pharmaceutical packaging information. Prescriptions with a dose titration were also identified (e.g., a low initial dose being increased over time to achieve a maintenance dose). Therefore, the initial dose was defined as the dose of the first period as indicated in a prescription, and the maintenance dose was defined as the highest dose as indicated in a prescription. If there was no dose titration in a prescription, the initial dose and the maintenance dose would be the same. The algorithm showed an accuracy of 92.0% in a validation sample of SSRI prescriptions.

A binary indicator of reduction in initial or maintenance dose of an SSRI prescription was defined as 50% reduction from 1 DDD/day based on two reasons: first, it is recommended that doses of citalopram, sertraline, and paroxetine should be reduced by half in patients with impaired kidney function, despite remarkable variation across different guidelines in terms of kidney function measures and dose adjustment schemes (Supplementary Table 1); second, a vast majority of the study population were prescribed an SSRI with a daily dose of either 0.5 or 1 DDD. According to the summary of product characteristics,^30^, ^31^ daily doses of 20 mg citalopram, 10 mg escitalopram, 20 mg fluoxetine, 20 mg paroxetine, or 50 mg sertraline are the recommended initial adult dosages, all of which correspond to 1 DDD/day (Supplementary Table 2).

### 2.4 Covariates

Covariates included age, sex, psychiatric diagnosis, and concurrent use of other central nervous system (CNS) medications. Psychiatric diagnoses (anxiety disorder, bipolar disorder, obsessive–compulsive disorder, and eating disorder) were identified by ICD‐10 codes from in‐patient or out‐patient care since 1997 until the index date. Other CNS medication use, defined as dispensed prescriptions within 1 year prior to the index date, was ascertained for antipsychotics; anxiolytics, hypnotics, and sedatives; attention‐deficit/hyperactivity disorder medication; drugs used in addictive disorders; opioids and pain medications; and antiepileptic drugs (Supplementary Table 3).

### 2.5 Statistical analyses

We examined the associations between eGFR categories and reductions in initial and maintenance dose among incident SSRI users using logistic regression models, with adjustment for age (in five‐year bands), sex, psychiatric diagnosis, and other CNS medication use. Results were reported as odds ratios (ORs) with 95% confidence intervals (CIs). To illustrate potential nonlinear associations, we investigated the relationships between eGFR and SSRI dose reduction using restricted cubic splines. We repeated the main analyses among those with a prior depression diagnosis. Stratified analyses by sex and age group (<65 vs. ≥65 years), and source of prescription (primary care, non‐psychiatric specialist care, or psychiatric care) were also performed. In a sensitivity analysis, the Modification of Diet in Renal Disease (MDRD) study equation was used for the calculation of eGFR.^32^ This is to explore the consistency of results should clinicians have applied this alternative equation when making dosage adjustments. We also investigated the associations between eGFR categories and dose reductions for citalopram and sertraline, the two most frequently used SSRIs, separately. All statistical analyses were performed using R software version 4.0.3 (R Foundation for Statistical Computing, Vienna, Austria).

## 3 RESULTS

**FIGURE 1:**
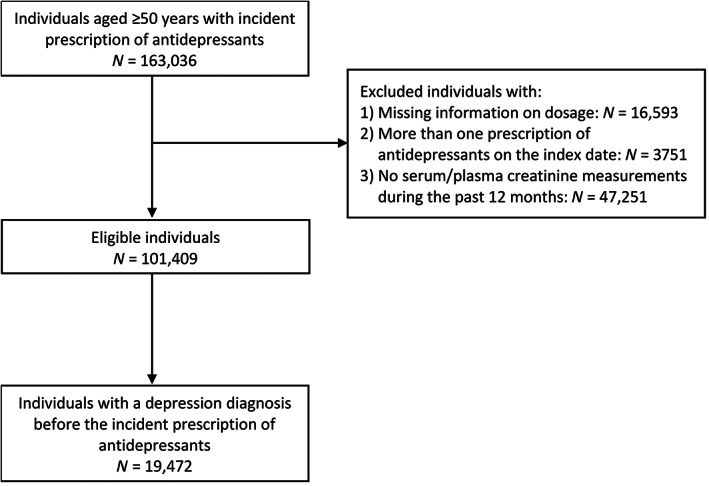
Flow chart of the sample selection

We identified 101 409 Stockholm residents aged ≥50 years started antidepressant treatment during the study period who had a recent creatinine test (Figure 1). Among them, 19 472 individuals had a prior diagnosis of depression. Lack of creatinine measurements was the main reason for exclusion (*n* = 47 251) and the excluded individuals were on average younger (mean age 65.1 years, SD 11.9) than included ones.

Among the included individuals, the mean age was 70.3 years (SD 12.3) and 59 910 (59.1%) were women. SSRIs accounted for more than half of the antidepressant prescriptions, and dose titration was found in one‐third of these prescriptions (Table 1). Citalopram was the most prescribed antidepressant (28.3%), followed by amitriptyline (20.7%) and mirtazapine (19.9%) (Supplementary Table 4). Most individuals received their prescriptions from primary care, with non‐psychiatric specialist care as the second most common source. The median eGFR was 81 ml/min/1.73 m^2^ (IQR 66–93). 11.4% of individuals had an eGFR 45–59 ml/min/1.73 m^2^, 5.3% had an eGFR 30–44 ml/min/1.73 m^2^, and 2.0% had an eGFR <30 ml/min/1.73 m^2^.

**TABLE 1:** Characteristics of the study population

	All incident antidepressant users			Incident antidepressant users with a depression diagnosis		
	Men (*n* = 41 499)	Women (*n* = 59 910)	Total (*n* = 101 409)	Men (*n* = 7587)	Women (*n* = 11 885)	Total (*n* = 19 472)
Age (years), mean (SD)	69.3 (11.8)	71.0 (12.6)	70.3 (12.3)	66.7 (11.6)	68.1 (12.3)	67.5 (12.1)
Type of antidepressant, *n* (%)						
SSRIs	21 287 (51.3)	30 999 (51.7)	52 286 (51.6)	5467 (72.1)	8747 (73.6)	14 214 (73.0)
SNRIs	2102 (5.1)	2308 (3.9)	4410 (4.3)	341 (4.5)	422 (3.6)	763 (3.9)
TCAs	7839 (18.9)	13 483 (22.5)	21 322 (21.0)	210 (2.8)	616 (5.2)	826 (4.2)
Other	10 271 (24.7)	13 120 (21.9)	23 391 (23.1)	1569 (20.7)	2100 (17.7)	3669 (18.8)
Source of prescription^a^, *n* (%)						
Primary care	23 796 (57.4)	37 810 (63.2)	61 606 (60.8)	5616 (74.1)	9428 (79.4)	15 044 (77.3)
Non‐psychiatric specialist care	14 081 (34.0)	18 644 (31.1)	32 725 (32.3)	758 (10.0)	1199 (10.1)	1957 (10.1)
Psychiatric care	3585 (8.6)	3400 (5.7)	6985 (6.9)	1208 (15.9)	1246 (10.5)	2454 (12.6)
Initial dose of SSRIs (DDDs/day)^b^, *n* (%)						
<0.5	256 (1.2)	745 (2.4)	1001 (1.9)	65 (1.2)	216 (2.5)	281 (2.0)
0.5	10 225 (48.0)	17 048 (55.0)	27 273 (52.2)	2465 (45.1)	4513 (51.6)	6978 (49.1)
1.0	10 296 (48.4)	12 662 (40.8)	22 958 (43.9)	2825 (51.7)	3862 (44.2)	6687 (47.0)
>1.0	510 (2.4)	544 (1.8)	1054 (2.0)	112 (2.0)	156 (1.8)	268 (1.9)
Maintenance dose of SSRIs (DDDs/day)^c^, *n* (%)						
<0.5	71 (0.3)	218 (0.7)	289 (0.6)	14 (0.3)	47 (0.5)	61 (0.4)
0.5	6209 (29.2)	11 355 (36.6)	17 564 (33.6)	1230 (22.5)	2552 (29.2)	3782 (26.6)
1.0	14 336 (67.3)	18 708 (60.4)	33 044 (63.2)	4065 (74.4)	5939 (67.9)	10 004 (70.4)
>1.0	671 (3.2)	718 (2.3)	1389 (2.7)	158 (2.9)	209 (2.4)	367 (2.6)
eGFR using CKD‐EPI equation (ml/min/1.73 m^2^), median (IQR)	83 (67–94)	80 (65–92)	81 (66–93)	85 (71–96)	83 (68–94)	84 (70–95)
eGFR using MDRD equation (ml/min/1.73 m^2^), median (IQR)	81 (67–95)	76 (63–90)	78 (64–92)	82 (70–96)	78 (66–91)	80 (67–93)
eGFR category based on CKD‐EPI equation (ml/min/1.73 m^2^), *n* (%)						
≥105	2114 (5.1)	2111 (3.5)	4225 (4.2)	439 (5.8)	506 (4.3)	945 (4.9)
90–104	11 654 (28.1)	14 963 (25.0)	26 617 (26.2)	2475 (32.6)	3535 (29.7)	6010 (30.9)
60–89	20 353 (49.0)	31 185 (52.1)	51 538 (50.8)	3668 (48.3)	6034 (50.8)	9702 (49.8)
45–59	4300 (10.4)	7290 (12.2)	11 590 (11.4)	613 (8.1)	1196 (10.1)	1809 (9.3)
30–44	2081 (5.0)	3296 (5.5)	5377 (5.3)	277 (3.7)	488 (4.1)	765 (3.9)
<30	997 (2.4)	1065 (1.8)	2062 (2.0)	115 (1.5)	126 (1.1)	241 (1.2)
Psychiatric diagnosis, *n* (%)						
Depression	7587 (18.3)	11 885 (19.8)	19 472 (19.2)	N/A	N/A	N/A
Anxiety disorder	4385 (10.6)	7925 (13.2)	12 310 (12.1)	892 (11.8)	1734 (14.6)	2626 (13.5)
Bipolar disorder	264 (0.6)	349 (0.6)	613 (0.6)	69 (0.9)	88 (0.7)	157 (0.8)
Obsessive–compulsive disorder	69 (0.2)	71 (0.1)	140 (0.1)	14 (0.2)	14 (0.1)	28 (0.1)
Eating disorder	15 (<0.1)	48 (0.1)	63 (0.1)	<10 (0.1)	15 (0.1)	19 (0.1)

Abbreviations: CKD‐EPI, chronic kidney disease epidemiology collaboration; DDDs, defined daily doses; eGFR, estimated glomerular filtration rate; MDRD, modification of diet in renal disease; SNRIs, serotonin–norepinephrine reuptake inhibitors; SSRIs, selective serotonin reuptake inhibitors; TCAs, tricyclic antidepressants.

^a^ Information on source of prescription was missing among 93 individuals.

^b^ 22 individuals with a prescribed dose between 0.5 and 1.0 DDDs/day (e.g., citalopram 15 mg) were included in the 0.5 DDDs/day group.

^c^ Drug titration was seen among 32.3% of individuals prescribed with SSRIs; 12 individuals with a prescription dosage between 0.5 and 1.0 DDDs/day were included in the 0.5 DDDs/day group.

Across all eGFR categories, SSRIs were the most commonly prescribed type of antidepressants (Supplementary Table 5). With lower eGFR, the percentages of SNRIs and TCAs use were lower, accompanied by a higher percentage of other antidepressants. Within SSRI users, citalopram was more often prescribed, while sertraline was less often used among individuals with lower eGFR.

Among incident antidepressant users with a depression diagnosis, the mean age was 67.5 years (SD 12.1) and 11 885 (61.0%) were women. SSRIs were prescribed among three quarters of this population, and amitriptyline use was much less common, as compared to the study sample irrespective of depression diagnosis. 9.3% of individuals had an eGFR 45–59 ml/min/1.73 m^2^, 3.9% had an eGFR 30–44 ml/min/1.73 m^2^, and 1.2% had an eGFR <30 ml/min/1.73 m^2^.

### 3.1 Kidney function and SSRI dose reduction

**FIGURE 2:**
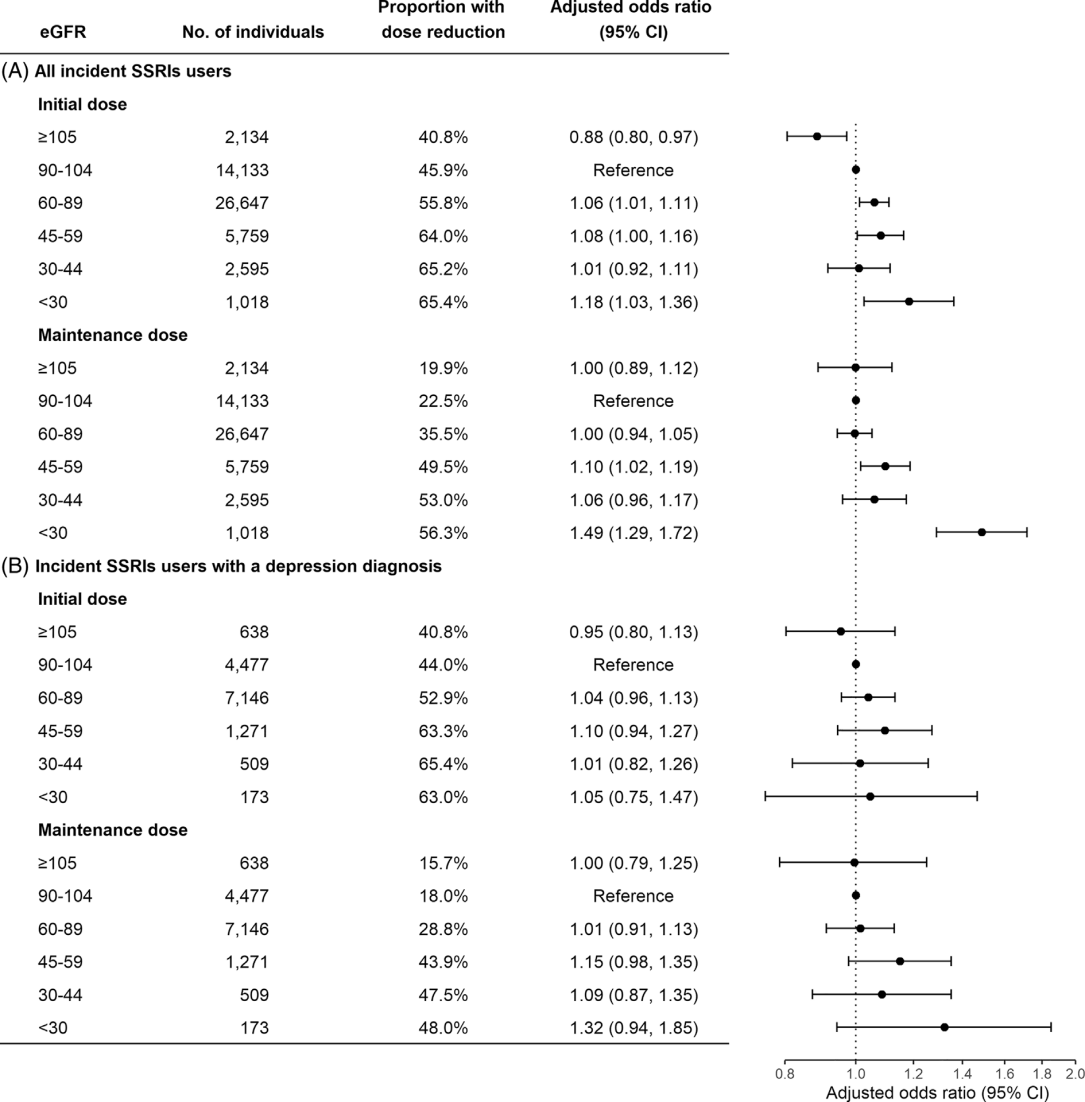
eGFR and SSRI dose reduction. Age, sex, psychiatric diagnosis, and other CNS medications use were adjusted for in the model. Abbreviations: eGFR, estimated glomerular filtration rate; SSRI, selective serotonin reuptake inhibitor

Reductions in initial dose and maintenance dose were observed among 54.1% and 34.1% of all incident SSRI users (Figure 2). The proportion of individuals receiving a reduced dose increased with lower eGFR for both initial and maintenance dose. Compared with eGFR category of 90–104 ml/min/1.73 m^2^, the proportions with a dose reduction increased from 45.9% to 65.4% for initial dose and from 22.5% to 56.3% for maintenance dose among patients with moderately to severely reduced eGFR (<30 ml/min/1.73 m^2^). After adjusting for covariates, for each lower eGFR category there were higher odds of reductions in initial dose and maintenance dose. The adjusted ORs (95% CIs) for reduced initial dose and reduced maintenance dose in those with moderately to severely reduced eGFR were 1.18 (1.03, 1.36) and 1.49 (1.29, 1.72), respectively (Supplementary Table 6). Analysis with restricted cubic splines showed non‐linear relationship between eGFR and the adjusted ORs for SSRI dose reduction (Supplementary Figure 1). Using eGFR of 90 ml/min/1.73 m^2^ as reference, lower eGFR did not appear to be associated with reduction of the initial SSRI dose. On the other hand, the odds of maintenance dose reduction became progressively higher with eGFR <50 ml/min/1.73 m^2^. When analyses were restricted to those with diagnosed depression, the associations between eGFR and SSRI dose reduction became non‐significant, whereas the pattern was similar to that found among all incident users of SSRIs (Figure 2).

### 3.2 Subgroup analyses

**FIGURE 3:**
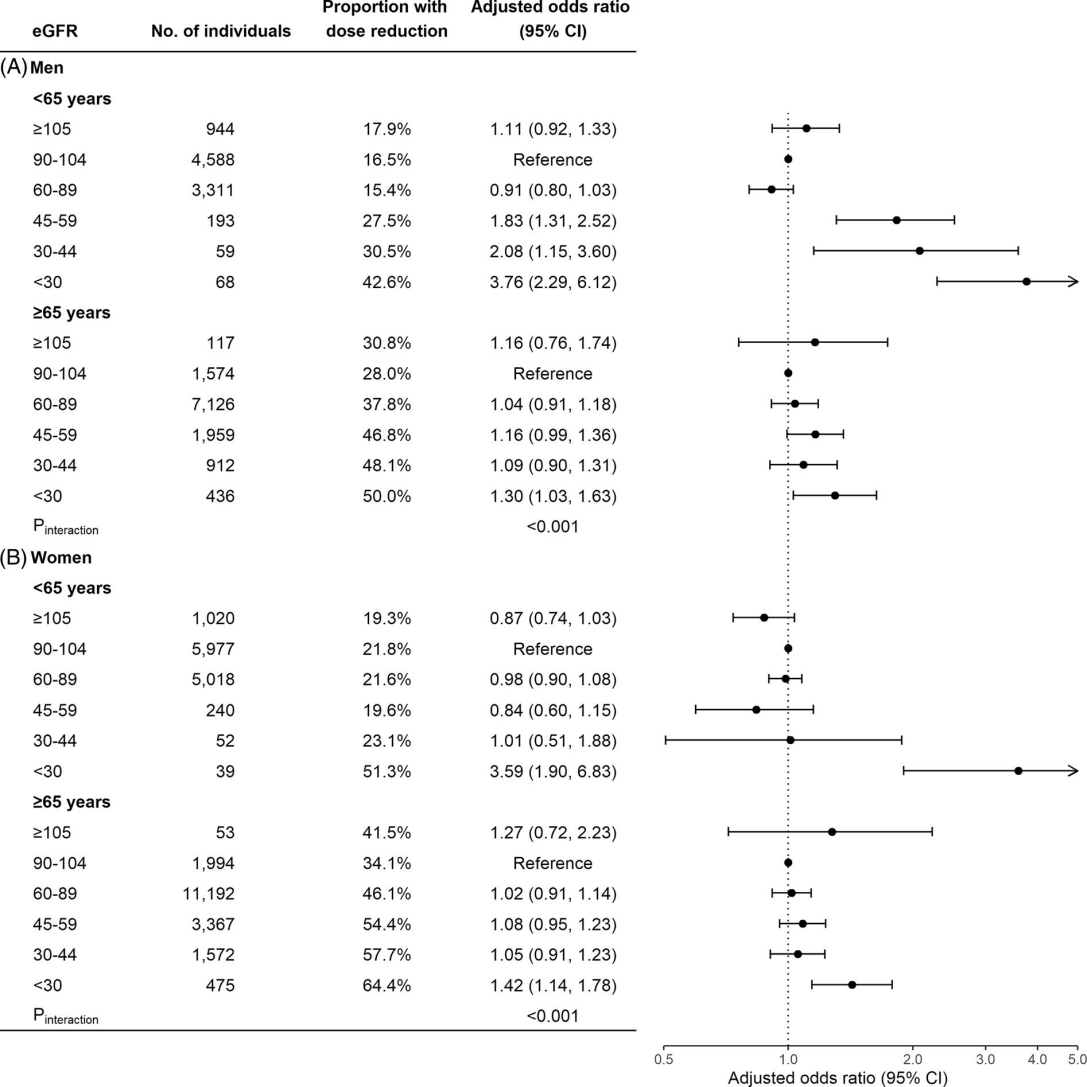
eGFR and reduction in maintenance dose of SSRIs, by sex and age groups. Age, psychiatric diagnosis, and other CNS medications use were adjusted for in the model. eGFR, estimated glomerular filtration rate; SSRIs, selective serotonin reuptake inhibitors

Older patients (≥65 years) showed higher proportions of reductions in initial dose (Supplementary Figure 2) and maintenance dose (Figure 3) compared to younger ones across eGFR categories. Nonetheless, when adjusting for covariates, the association between eGFR and SSRI dose reduction was more pronounced among individuals aged 50–64 years, than among individuals aged ≥65 years (*P*_interaction_ <0.001). In individuals aged 50–64 years, the adjusted ORs (95% CIs) of moderately to severely reduced eGFR with maintenance dose reduction were 3.76 (2.29, 6.12) in men and 3.59 (1.90, 6.83) in women. In contrast, the corresponding ORs (95% CIs) among individuals aged ≥65 years were 1.30 (1.03, 1.63) in men and 1.42 (1.14, 1.78) in women, respectively.

**FIGURE 4:**
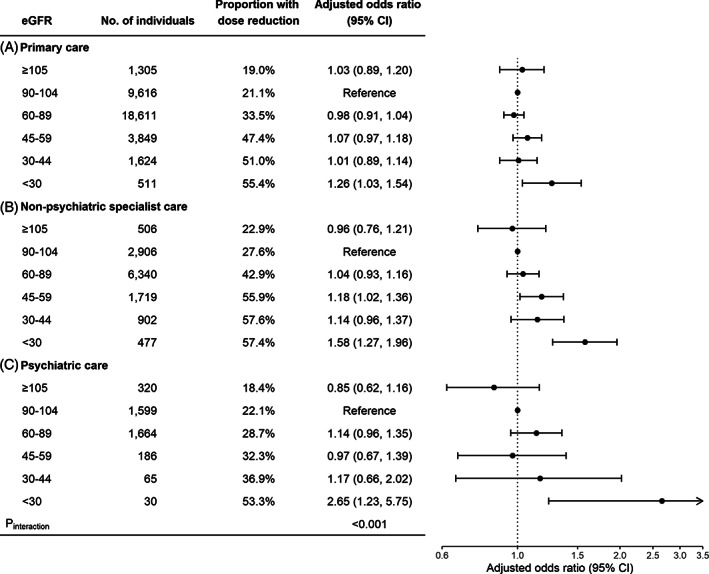
eGFR and reduction in maintenance dose of SSRIs, by source of prescription. Age, sex, psychiatric diagnosis, and other CNS medications use were adjusted for in the model. eGFR, estimated glomerular filtration rate; SSRIs, selective serotonin reuptake inhibitors

In the stratified analysis by source of prescription, overall higher proportions of reductions in initial dose (Supplementary Figure 3) and maintenance dose (Figure 4) were seen among individuals who received their prescriptions from primary care and non‐psychiatric specialist care, compared with those receiving prescriptions from psychiatric care. However, there was a stronger association between lower eGFR and SSRI dose reduction within psychiatric care setting, with the adjusted ORs (95% CIs) of moderately to severely reduced eGFR being 1.85 (0.87, 4.11) for initial dose reduction and 2.65 (1.23, 5.75) for maintenance dose reduction (*P*_interaction_ <0.001).

### 3.3 Sensitivity analyses

Sensitivity analyses using the MDRD study equation to calculate eGFR produced similar results (Supplementary Figure 4). Compared with the main analyses for any SSRI prescription, we observed a higher proportion of dose reduction for citalopram and a lower proportion for sertraline, while the patterns of association between eGFR and dose reduction were similar (Supplementary Figure 5).

## 4 DISCUSSION

In this population‐based study, we investigated the association between kidney function and SSRI dosing for middle‐aged and older patients in routine clinical practice. This study shows that individuals with lower kidney function were more likely to be prescribed a reduced SSRI dose, with regard to the initial as well as the maintenance dose, than individuals with normal kidney function. However, still two fifths of patients with moderately to severely decreased kidney function (i.e., eGFR <30 ml/min/1.73 m^2^) received an SSRI prescription without dose reduction, leaving them at potential risk of adverse drug reactions. The association between reduced kidney function and SSRI dose reduction was more evident among middle‐aged population and within psychiatric care.

Our study demonstrated that reduced kidney function and increased age were both associated with SSRI dose reduction. First, as one would expect, dose reduction for SSRIs was more common among the older age group, reflecting age as one of the major considerations for dose adjustment in the prescribing practice.^33^ For example, the Swedish national guideline recommends lower doses of citalopram and escitalopram in individuals ≥65 years of age because of an increased risk of QT prolongation.^34^ Second, the proportion of SSRI dose reduction increased with lower eGFR; about 60% of individuals with an eGFR <30 ml/min/1.73 m^2^ were prescribed a reduced dose of SSRIs. On the other hand, it means that two out of five patients with moderate to severe loss of kidney function received SSRIs without dose reduction and were predisposed to potential adverse drug reactions. Third, this study further indicated that individuals with lower eGFR were more likely to receive an SSRI prescription with reduced dose, in addition to the influence of age. However, the adjusted odds ratios were of modest magnitude, suggesting that kidney function was not always considered when making decisions on antidepressant prescription.

Our subgroup analyses revealed that age modified the relationship between kidney function and SSRI dose reduction. Although the absolute proportion of SSRI dose reduction was higher among older adults, the association between lower eGFR and SSRI dose reduction was less pronounced in this age category. A larger proportion of older patients were prescribed SSRIs with reduced dose regardless of their kidney function, and as a consequence, we may not anticipate a strong association between kidney function and SSRI dose reduction in the older age group. Nevertheless, no matter whether loss of kidney function is due to older age or underlying diseases, dosages should be adjusted accordingly for certain drugs to avoid the risk of adverse drug reactions.

Few studies have evaluated the implementation of SSRI dose adjustments in the routine care of patients with kidney disease and depression. One previous study in the UK primary care setting found no clear evidence that antidepressants were started at a reduced dose in patients with CKD (defined as eGFR <60 ml/min/1.73 m^2^),^22^ but they only compared the median initial dose between patients with and without CKD. Our study instead assessed the whole eGFR spectrum and showed that eGFR was not associated with reduction of the initial SSRI dose among individuals who received a prescription from primary care, whereas eGFR was more strongly associated with a reduced maintenance dose. Furthermore, our study demonstrated a stronger association among those whose treatment was initiated in psychiatric care. However, it should be noted that the proportions of dose reduction in patients with low eGFR were similar regardless of levels of care. This larger effect size does not necessarily mean that psychiatric prescribers are more likely to take eGFR into account when determining drug dosing; on the contrary, it could indicate that patients with normal kidney function are more susceptible to be treated with suboptimal SSRI doses by primary care physicians.^35^

Existing guidelines highlight that a lower dose regimen for SSRIs should be applied to patients with CKD; at the same time, other recent studies instead point to the issue of SSRI doses in clinical practice often being too low to elicit a maximum antidepressant effect.^35^, ^36^ In line with these findings, we noticed that only a small fraction of individuals with normal kidney function received a prescribed daily dose higher than 1 DDD—the lower bound of maximum effective dose corresponding to >1 DDD for citalopram, escitalopram and sertraline, but equaling 1 DDD for fluoxetine and paroxetine.^37^ Taken together, there are clinical implications for the optimization of antidepressant treatment. Optimal use of SSRIs requires making individualized treatment plans according to patients' profiles and needs, and kidney function is one of the important factors that should be taken into account, owing to the commonness of creatinine test in routine clinical practice. On the one hand, to prevent potential adverse effects, lower SSRI doses are supposed to be prescribed to individuals with reduced kidney function. On the other hand, for individuals with normal kidney function, a higher SSRI dose that could exert a maximum antidepressant effect should be considered to combat the symptoms of depression more effectively.

Despite the high prevalence of depression and the reported associations between comorbid depression and poor outcomes among patients with CKD, few data exist regarding the efficacy and safety of antidepressant treatment in this patients group.^38^ Randomized clinical trials found that the sertraline group compared to the placebo group did not show a significant improvement in depressive symptoms, but experienced a higher incidence of adverse events.^39^, ^40^ Furthermore, long‐term use of antidepressants may predispose patients to adverse drug reactions due to the potential accumulation of toxic metabolites and increased risk of drug–drug interactions. Observational studies have documented several adverse health consequences associated with SSRI use in patients with CKD, such as hip fracture, gastrointestinal bleeding, and sudden cardiac death due to QT prolongation.^41^, ^42^, ^43^ Clinical guidelines for the treatment of depression generally suggest careful attention to kidney function among elderly patients,^8^, ^9^, ^10^, ^11^ but there is considerable variation in dose adjustment recommendations for antidepressants. There is no prior research on how prescribers might choose from these recommendations. Further studies are needed to support evidence‐based decisions for the treatment of depression in patients with CKD, and to evaluate potential adverse drug reactions derived from high dosage.

The main strength of our study was the unique linkage of laboratory tests with population‐based health registers. However, several limitations should be taken into consideration. First, a single serum creatinine measure may not be enough to determine the presence of CKD, but reflects the role of the most recent assessment of kidney function on SSRI dosing, and this is surely the information at hand for the physicians. Second, nearly 30% of new users of antidepressants were excluded due to lack of creatinine measurements; these were younger and likely to be healthier than those included, so the results may not generalize to younger population. Nevertheless, this observation may remind prescribers of the need to evaluate kidney function in their patients and consider this information when prescribing SSRIs. Third, although the algorithm used to estimate the prescribed daily dose has a high accuracy, misclassifications of the prescribed dose may be inevitable. Further, the prescribed daily dose was missing among 10% of the prescriptions, and these were excluded from the analyses. Assuming that the missingness occurred at random, the observed association would have been diluted. Fourth, we did not have information on the indications for antidepressant prescriptions, as they are not systematically recorded. Nevertheless, similar patterns of association were observed in individuals with and without a diagnosis of depression. Fifth, only the first SSRI prescription was analyzed. While it is possible that dose reductions in the following prescriptions were missed, we see it as unlikely that physicians would prescribe a higher dose to patients with reduced kidney function in the beginning and gradually decrease the dose. The current study examined only the prevalence of dose adjustment, and not whether certain drugs were avoided in those with low kidney function. Finally, prescribing practices may vary in different healthcare settings and across countries. Thus, extrapolation to other settings should be done with caution.

In conclusion, our study shows that lower kidney function was moderately associated with reduction in prescribed SSRI dose in addition to age, with this association being more evident in the middle‐aged individuals and among treatments initiated in psychiatric care. There exists a gap between current recommendations and routine prescribing practice; kidney function should receive more attention when prescribing SSRIs. Still, there is a paucity of evidence on efficacy and safety of antidepressants in patients with CKD. More studies are warranted to provide better evidence‐based treatments for patients with CKD and mood disorders.

## AUTHOR CONTRIBUTIONS

Nanbo Zhu performed the statistical analysis, interpreted the results, and drafted the manuscript. Alexander Lisinski, Kristina Johnell, and Hong Xu interpreted the results. Tyra Lagerberg analyzed and interpreted the results. Juan Jesús Carrero acquired the data and interpreted the results. Zheng Chang designed the study and interpreted the results. All authors reviewed the manuscript for critical content and approved the final version of the manuscript.

## FUNDING INFORMATION

This study is supported by grants from the Swedish Research Council (2018–02213; 2019–01059) and Loo and Hans Osterman Foundation for Medical Research.

## CONFLICT OF INTEREST

The authors declare no conflict of interest.

## ETHICS STATEMENT

This study was approved by regional ethics review boards in Stockholm and adhered to the Declaration of Helsinki.

## PREVIOUS PRESENTATION

The abstract has been presented at the 13th annual Nordic PharmacoEpidemiological Network meeting in Stockholm on 11 November 2021.

## Supporting information


**Supplementary table 1** Summary of recommendations on SSRI dosing in patients with impaired kidney function
**Supplementary table 2**. Information on SSRI dosing in people with normal kidney function
**Supplementary table 3**. ICD‐10 codes used to define psychiatric diagnosis and ATC codes used to define concurrent use of CNS medications
**Supplementary table 4.** Distribution of incident antidepressant prescriptions in the study population
**Supplementary table 5.** Type of antidepressants according to eGFR categories
**Supplementary table 6.** Associations between covariates and SSRI dose reduction
**Supplementary figure 1.** Association between eGFR and SSRI dose reduction using restricted cubic splines
**Supplementary figure 2.** eGFR and reduction in initial dose of SSRIs, by sex and age groups
**Supplementary figure 3.** eGFR and reduction in initial dose of SSRIs, by source of prescription
**Supplementary figure 4.** eGFR and SSRI dose reduction using the MDRD study equation
**Supplementary figure 5.** eGFR and dose reductions for citalopram and sertraline

## Data Availability

Qualified researchers can get access to the data underlying the reported results provided that the request is compliant with Swedish data protection laws and the Swedish Ethical Review Act.
