# Supplementary material for: Kidney function and prescribed dose in middle‐aged and older patients starting selective serotonin reuptake inhibitors
Source: Pharmacoepidemiol Drug Saf. 2022 Aug 10;31(10):1091–101. doi: 10.1002/pds.5515 (PMC9545078; doi:10.1002/pds.5515)
Supplement: Supplementary file 1 — Supplementary table 1 Summary of recommendations on SSRI dosing in patients with impaired kidney function Supplementary table 2. Information on SSRI dosing in people with normal kidney function Supplementary table 3. ICD‐10 codes used to define psychiatric diagnosis and ATC codes used to define concurrent use of CNS medications Supplementary table 4. Distribution of incident antidepressant prescriptions in the study population Supplementary table 5. Type of antidepressants according to eGFR categories Supplementary table 6. Associations between covariates and SSRI dose reduction Supplementary figure 1. Association between eGFR and SSRI dose reduction using restricted cubic splines Supplementary figure 2. eGFR and reduction in initial dose of SSRIs, by sex and age groups Supplementary figure 3. eGFR and reduction in initial dose of SSRIs, by source of prescription Supplementary figure 4. eGFR and SSRI dose reduction using the MDRD study equation Supplementary figure 5. eGFR and dose reductions for citalopram and sertraline [file PDS-31-1091-s001.docx]

**Supplementary material**

**Kidney function and prescribed dose in middle-aged and older patients starting selective serotonin reuptake inhibitors**

Nanbo Zhu, Alexander Lisinski, Tyra Lagerberg, Kristina Johnell, Hong Xu,

Juan Jesús Carrero, Zheng Chang

[**Supplementary table 1.** Summary of recommendations on SSRI dosing in patients with impaired kidney function 2](#_Toc107576130)

[**Supplementary table 2.** Information on SSRI dosing in people with normal kidney function 3](#_Toc107576131)

[**Supplementary table 3.** ICD-10 codes used to define psychiatric diagnosis and ATC codes used to define concurrent use of CNS medications 4](#_Toc107576132)

[**Supplementary table 4.** Distribution of incident antidepressant prescriptions in the study population 5](#_Toc107576133)

[**Supplementary table 5.** Type of antidepressants according to eGFR categories 6](#_Toc107576134)

[**Supplementary table 6.** Associations between covariates and SSRI dose reduction 7](#_Toc107576135)

[**Supplementary figure 1.** Association between eGFR and SSRI dose reduction using restricted cubic splines 8](#_Toc107576136)

[**Supplementary figure 2.** eGFR and reduction in initial dose of SSRIs, by sex and age groups 9](#_Toc107576137)

[**Supplementary figure 3.** eGFR and reduction in initial dose of SSRIs, by source of prescription 10](#_Toc107576138)

[**Supplementary figure 4.** eGFR and SSRI dose reduction using the MDRD study equation 11](#_Toc107576139)

[**Supplementary figure 5.** eGFR and dose reductions for citalopram and sertraline 12](#_Toc107576140)

**Supplementary table 1.** Summary of recommendations on SSRI dosing in patients with impaired kidney function

|  | **Citalopram** | **Escitalopram** | **Fluoxetine** | **Paroxetine** | **Sertraline** |
| --- | --- | --- | --- | --- | --- |
| The Swedish Summary of Product Characteristics^1^ | CrCl <30 ml/min *Avoid* | CrCl <30 ml/min  *Use with caution* | – | CrCl <30 ml/min  *Restrict to the lower end of the dosage spectra* | – |
| European Renal Best Practice Recommendations^2^ | – | – | – | eGFR <60 ml/min  *Initial dose 10 mg/day* | eGFR <15 ml/min *Initial dose 25 mg/day* |
| Hedayati SS et al.^3^ | eGFR <60 ml/min *Initial dose 10 mg/day* eGFR <20 ml/min  *Not recommended* | eGFR <30 ml/min  *Use with caution* | – | eGFR <60 ml/min  *Initial dose 10 mg/day* | – |
| VA/DoD Clinical Practice Guideline^4^ | CrCl <20 ml/min *Initial dose 10 mg/day* | CrCl <20 ml/min *Initial dose 5 mg/day* | Renal impairment  *Reduce dose or frequency* | Renal impairment  *Initial dose 10 mg/day* | – |
| The Renal Drug Handbook^5^ | eGFR <10 ml/min  *Use with caution* | eGFR <30 ml/min *Start with a low dose* | eGFR <10 ml/min  *Reduce dose or frequency* | – | – |
| The Maudsley Prescribing Guidelines in Psychiatry^6^ | eGFR <10 ml/min  *Use with caution* | eGFR <30 ml/min *Start with a low dose* | eGFR <20 ml/min  *Reduce dose or frequency* | eGFR <30 ml/min  *Initial dose 10 mg/day* | – |

Abbreviations: CKD, chronic kidney disease; CrCl, creatinine clearance; eGFR, estimated glomerular filtration rate; SSRI, selective serotonin reuptake inhibitor.

**Supplementary table 2.** Information on SSRI dosing in people with normal kidney function

| **Drug name** | **Initial adult dosage** | **Usual adult dosage** | **Defined Daily Dose** |
| --- | --- | --- | --- |
| Citalopram | 20 mg/d | 20-40 mg/d | 20 mg |
| Escitalopram | 10 mg/d | 10-20 mg/d | 10 mg |
| Fluoxetine | 20 mg/d | 20-60 mg/d | 20 mg |
| Paroxetine | 20 mg/d | 20-50 mg/d | 20 mg |
| Sertraline | 50 mg/d | 50-200 mg/d | 50 mg |

Source of information is from the Swedish summaries of product characteristics, available at https://www.fass.se/.

**Supplementary table 3.** ICD-10 codes used to define psychiatric diagnosis and ATC codes used to define concurrent use of CNS medications

| **Psychiatric diagnosis** | **ICD-10 codes** |
| --- | --- |
| Anxiety disorder | F40, F41 |
| Bipolar disorder | F31 |
| Obsessive-compulsive disorder | F42 |
| Eating disorder | F50 |
|  |  |
| **Concurrent use of CNS medications** | **ATC codes** |
| Antipsychotics | N05A |
| Anxiolytics, hypnotics, and sedatives | N05B, N05C |
| Attention-deficit/hyperactivity disorder medication | N06B |
| Drugs used in addictive disorders | N07B |
| Opioids and pain medications | N02A |
| Antiepileptic drugs | N03A |

Abbreviations: ATC, Anatomical Therapeutic Chemical Classification system; CNS, central nervous system; ICD, International Statistical Classification of Diseases and Related Health Problems.

**Supplementary table 4.** Distribution of incident antidepressant prescriptions in the study population

| **ATC code** | **Drug name** | **Frequency** | **Proportion (%)** |
| --- | --- | --- | --- |
| **SSRIs** | | | |
| N06AB03 | Fluoxetine | 1,456 | 1.4 |
| N06AB04 | Citalopram | 28,720 | 28.3 |
| N06AB05 | Paroxetine | 689 | 0.7 |
| N06AB06 | Sertraline | 12,015 | 11.8 |
| N06AB10 | Escitalopram | 9,399 | 9.3 |
| **SNRIs** | | | |
| N06AX16 | Venlafaxine | 1,222 | 1.2 |
| N06AX21 | Duloxetine | 3,188 | 3.1 |
| **TCAs** | | | |
| N06AA04 | Clomipramine | 253 | 0.2 |
| N06AA09 | Amitriptyline | 20,972 | 20.7 |
| N06AA10 | Nortriptyline | 82 | 0.1 |
| **Others** | | | |
| N06AG02 | Moclobemide | 28 | <0.1 |
| N06AX03 | Mianserin | 391 | 0.4 |
| N06AX11 | Mirtazapine | 20,187 | 19.9 |
| N06AX12 | Bupropion | 2,401 | 2.4 |
| N06AX18 | Reboxetine | 40 | <0.1 |
| N06AX22 | Agomelatine | 193 | 0.2 |
| N06AX26 | Vortioxetine | 151 | 0.1 |

Abbreviations: ATC, Anatomical Therapeutic Chemical Classification system; SNRIs, serotonin–norepinephrine reuptake inhibitors; SSRIs, selective serotonin reuptake inhibitors; TCAs, tricyclic antidepressants.

Prescriptions with frequency less than 10 have been omitted to preserve patient privacy.

**Supplementary table 5.** Type of antidepressants according to eGFR categories

|  | **All incident antidepressant users** | | | | | |  | **Incident antidepressant users with a depression diagnosis** | | | | | |
| --- | --- | --- | --- | --- | --- | --- | --- | --- | --- | --- | --- | --- | --- |
|  | ≥105  (n=4,225) | 90-104  (n=26,617) | 60-89  (n=51,538) | 45-59  (n=11,590) | 30-44  (n=5,377) | <30  (n=2,062) |  | ≥105  (n=945) | 90-104  (n=6,010) | 60-89  (n=9,702) | 45-59  (n=1,809) | 30-44  (n=765) | <30  (n=241) |
| SSRIs (%) | 50.5 | 53.1 | 51.7 | 49.7 | 48.3 | 49.4 |  | 67.5 | 74.5 | 73.7 | 70.3 | 66.5 | 71.8 |
| Citalopram | 24.1 | 25.2 | 29.0 | 31.8 | 31.1 | 33.8 |  | 32.1 | 36.6 | 41.5 | 44.4 | 44.1 | 49.0 |
| Escitalopram | 8.9 | 9.4 | 9.4 | 9.0 | 8.8 | 7.6 |  | 9.9 | 12.4 | 12.0 | 12.2 | 11.8 | 10.8 |
| Fluoxetine | 2.1 | 2.2 | 1.3 | 0.7 | 0.5 | 0.4 |  | 2.0 | 2.5 | 1.6 | 0.7 | 0.7 | <0.1 |
| Paroxetine | 1.0 | 0.9 | 0.6 | 0.4 | 0.4 | 0.3 |  | 1.0 | 0.7 | 0.7 | 0.5 | <0.1 | 0.8 |
| Sertraline | 14.3 | 15.3 | 11.4 | 7.9 | 7.5 | 7.2 |  | 22.5 | 22.2 | 17.8 | 12.5 | 10.1 | 11.2 |
| SNRIs (%) | 5.1 | 5.4 | 4.3 | 3.3 | 2.4 | 1.6 |  | 4.9 | 4.8 | 3.7 | 2.6 | 2.1 | 0.4 |
| TCAs (%) | 23.0 | 22.5 | 21.1 | 19.3 | 16.9 | 15.5 |  | 6.9 | 5.1 | 3.8 | 3.5 | 2.5 | 2.5 |
| Others (%) | 21.4 | 19.0 | 22.9 | 27.6 | 32.4 | 33.6 |  | 20.7 | 15.5 | 18.9 | 23.7 | 28.9 | 25.3 |

Abbreviations: eGFR, estimated glomerular filtration rate; SNRIs, serotonin–norepinephrine reuptake inhibitors; SSRIs, selective serotonin reuptake inhibitors; TCAs, tricyclic antidepressants.

**Supplementary table 6.** Associations between covariates and SSRI dose reduction

|  | **Initial dose** | **Maintenance dose** |
| --- | --- | --- |
| eGFR category (mL/min/1.73 m^2^) |  |  |
| ≥105 | 0.88 (0.80, 0.97) | 1.00 (0.89, 1.12) |
| 90–104 | 1.00 | 1.00 |
| 60–89 | 1.06 (1.01, 1.11) | 1.00 (0.94, 1.05) |
| 45–59 | 1.08 (1.00, 1.16) | 1.10 (1.02, 1.19) |
| 30–44 | 1.01 (0.92, 1.11) | 1.06 (0.96, 1.17) |
| <30 | 1.18 (1.03, 1.36) | 1.49 (1.29, 1.72) |
| Female | 1.32 (1.27, 1.37) | 1.36 (1.30, 1.41) |
| Age category (years) |  |  |
| 50–54 | 1.00 | 1.00 |
| 50–59 | 1.04 (0.98, 1.11) | 1.01 (0.93, 1.10) |
| 60–64 | 1.07 (1.00, 1.14) | 1.18 (1.09, 1.29) |
| 65–69 | 1.22 (1.14, 1.31) | 1.63 (1.50, 1.77) |
| 70–74 | 1.42 (1.32, 1.52) | 2.16 (1.99, 2.35) |
| 75–79 | 1.87 (1.73, 2.01) | 3.15 (2.90, 3.43) |
| 80–84 | 2.31 (2.14, 2.50) | 4.27 (3.92, 4.65) |
| 85–89 | 3.17 (2.91, 3.46) | 6.39 (5.83, 7.01) |
| ≥90 | 3.91 (3.50, 4.38) | 9.14 (8.16, 10.25) |
| Psychiatric diagnosis |  |  |
| Anxiety disorder | 1.04 (0.99, 1.09) | 0.93 (0.88, 0.98) |
| Bipolar disorder | 1.01 (0.79, 1.30) | 1.00 (0.76, 1.31) |
| Obsessive-compulsive disorder | 1.02 (0.69, 1.50) | 1.10 (0.70, 1.70) |
| Eating disorder | 0.80 (0.41, 1.51) | 1.07 (0.50, 2.15) |
| CNS medication use |  |  |
| Opioids and pain medications | 0.96 (0.92, 1.00) | 0.99 (0.95, 1.04) |
| Antiepileptic drugs | 0.91 (0.84, 1.00) | 1.30 (1.19, 1.43) |
| Antipsychotics | 1.04 (0.95, 1.14) | 1.46 (1.32, 1.61) |
| Anxiolytics, hypnotics, and sedatives | 0.96 (0.92, 0.99) | 0.85 (0.82, 0.89) |
| ADHD medication | 1.17 (0.85, 1.61) | 1.74 (1.24, 2.43) |
| Drugs used in addictive disorders | 0.97 (0.86, 1.09) | 1.01 (0.88, 1.15) |

Abbreviations: ADHD, Attention-deficit/hyperactivity disorder; CNS, central nervous system; eGFR, estimated glomerular filtration rate.


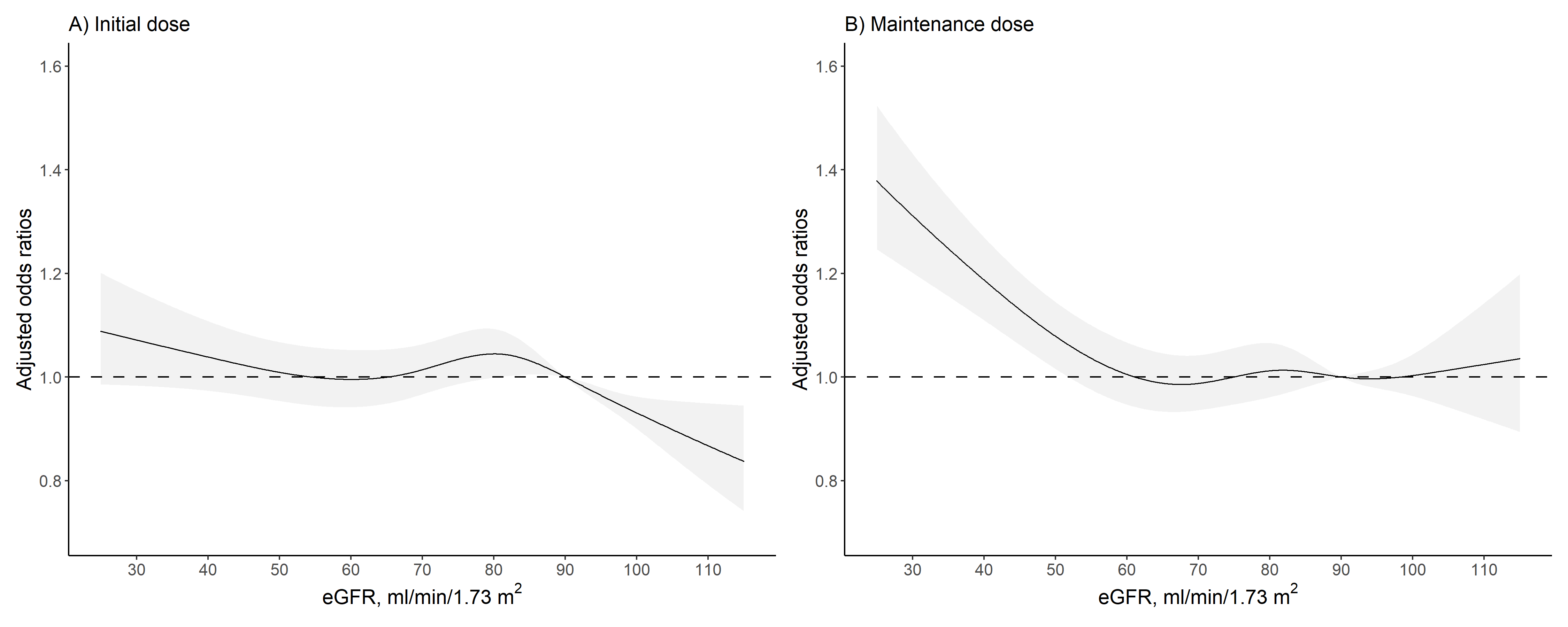


**Supplementary figure 1.** Association between eGFR and SSRI dose reduction using restricted cubic splines

Abbreviations: eGFR, estimated glomerular filtration rate; SSRI, selective serotonin reuptake inhibitor.

Odds ratios were modelled using restricted cubic splines with 5 knots at 5, 27.5, 50, 72.5 and 95 percentiles (which corresponded to eGFR of 41, 68, 81, 92 and 104 mL/min/1.73 m^2^). The eGFR of 90 mL/min/1.73 m^2^ was used as reference. Age, sex, psychiatric diagnosis, and use of other CNS medications were adjusted for in the model.


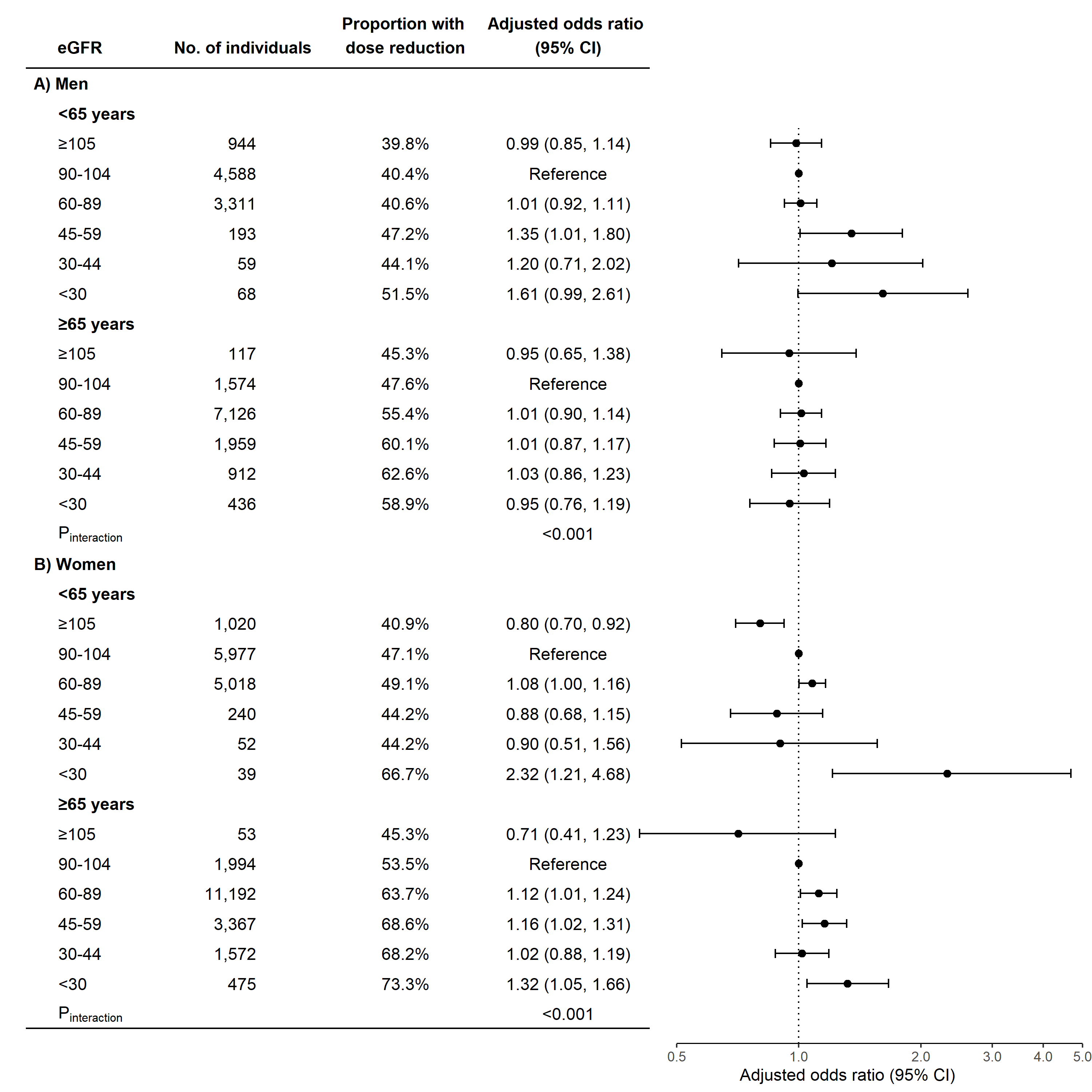


**Supplementary figure 2.** eGFR and reduction in initial dose of SSRIs, by sex and age groups

Abbreviations: eGFR, estimated glomerular filtration rate; CI, confidence interval; SSRIs, selective serotonin reuptake inhibitors.

Age, psychiatric diagnosis, and use of other CNS medications were adjusted for in the model.


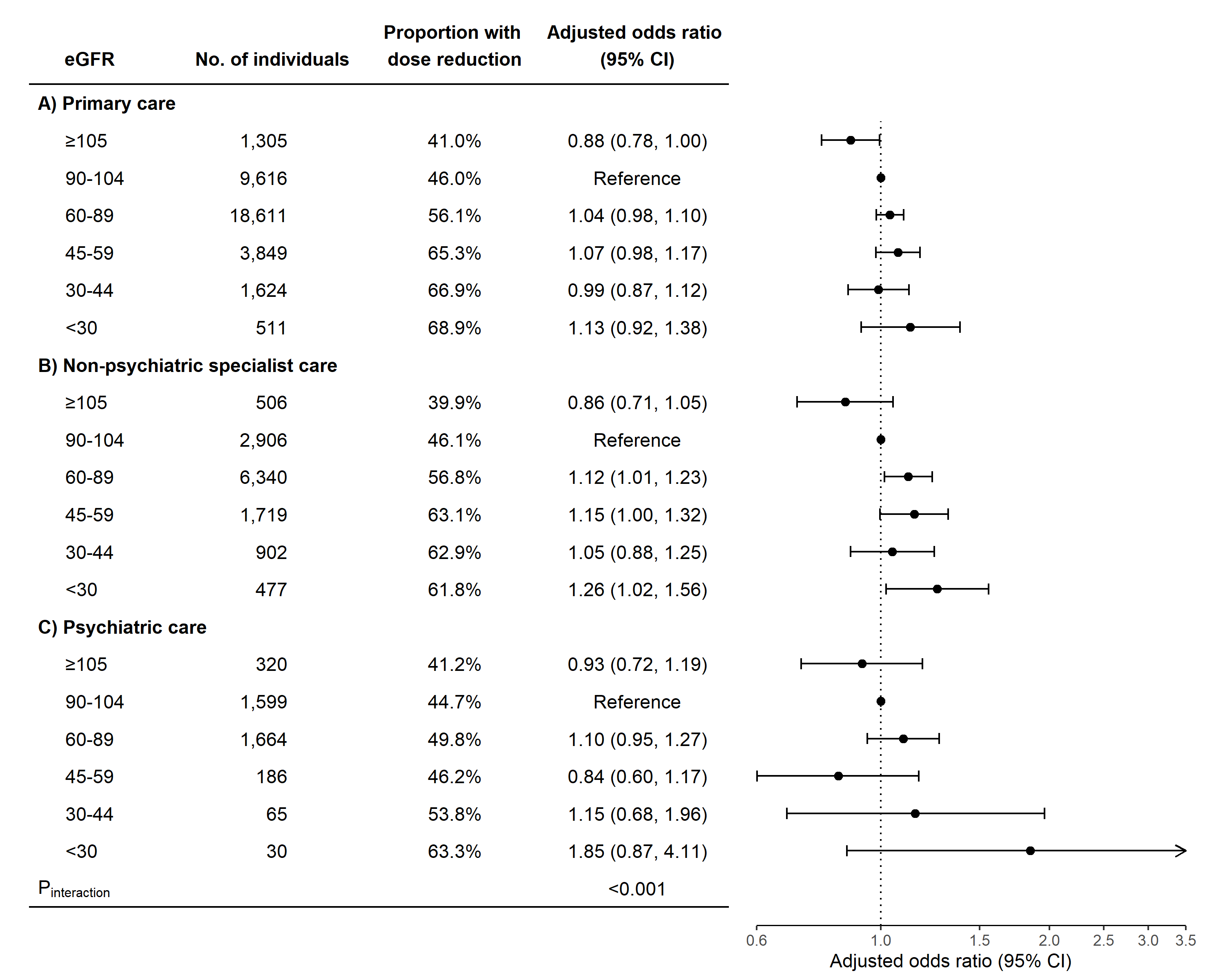


**Supplementary figure 3.** eGFR and reduction in initial dose of SSRIs, by source of prescription

Abbreviations: eGFR, estimated glomerular filtration rate; CI, confidence interval; SSRIs, selective serotonin reuptake inhibitors.

Age, sex, psychiatric diagnosis, and use of other CNS medications were adjusted for in the model.


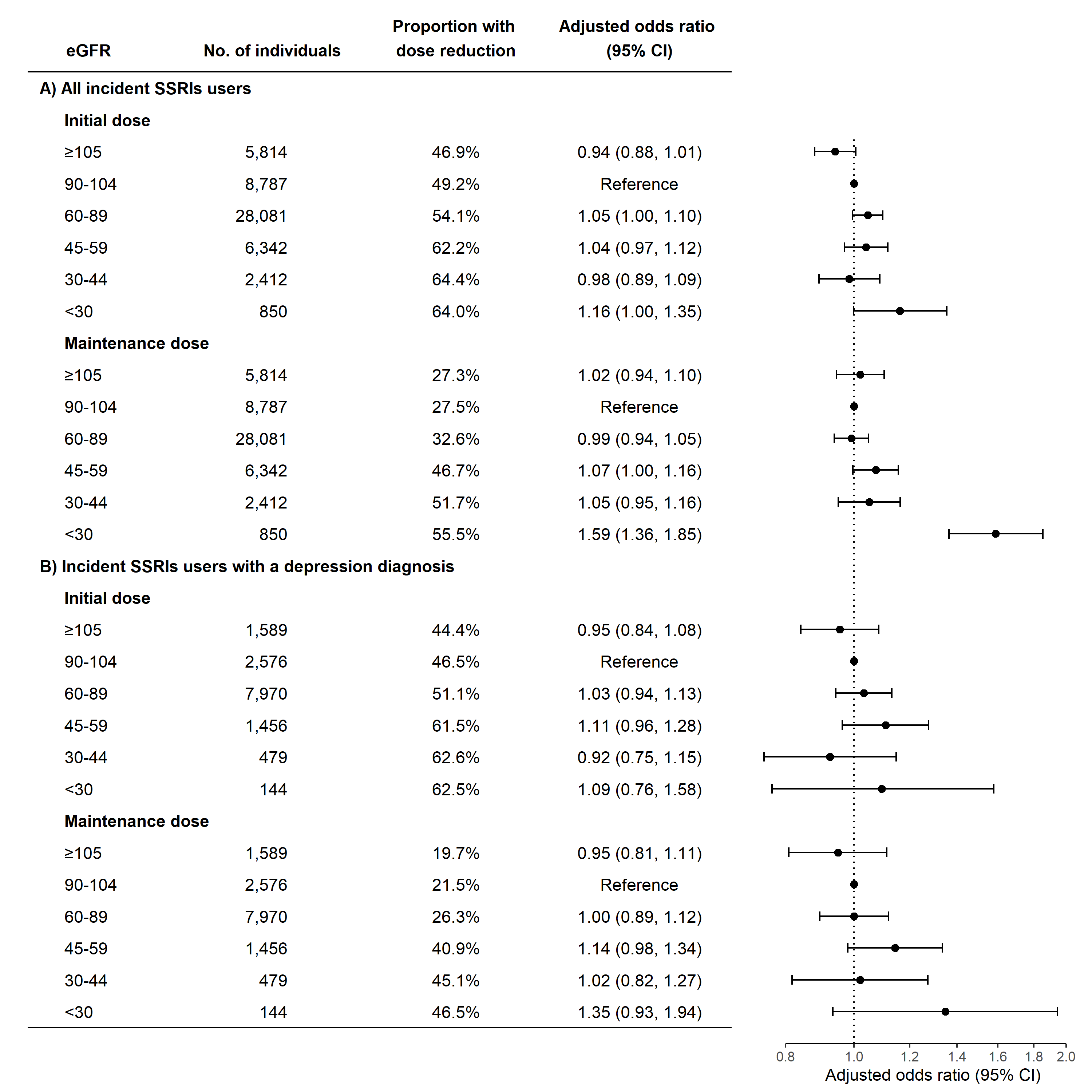


**Supplementary figure 4.** eGFR and SSRI dose reduction using the MDRD study equation

Abbreviations: eGFR, estimated glomerular filtration rate; CI, confidence interval; MDRD, Modification of Diet in Renal Disease; SSRI, selective serotonin reuptake inhibitor.

Age, sex, psychiatric diagnosis, and use of other CNS medications were adjusted for in the model.


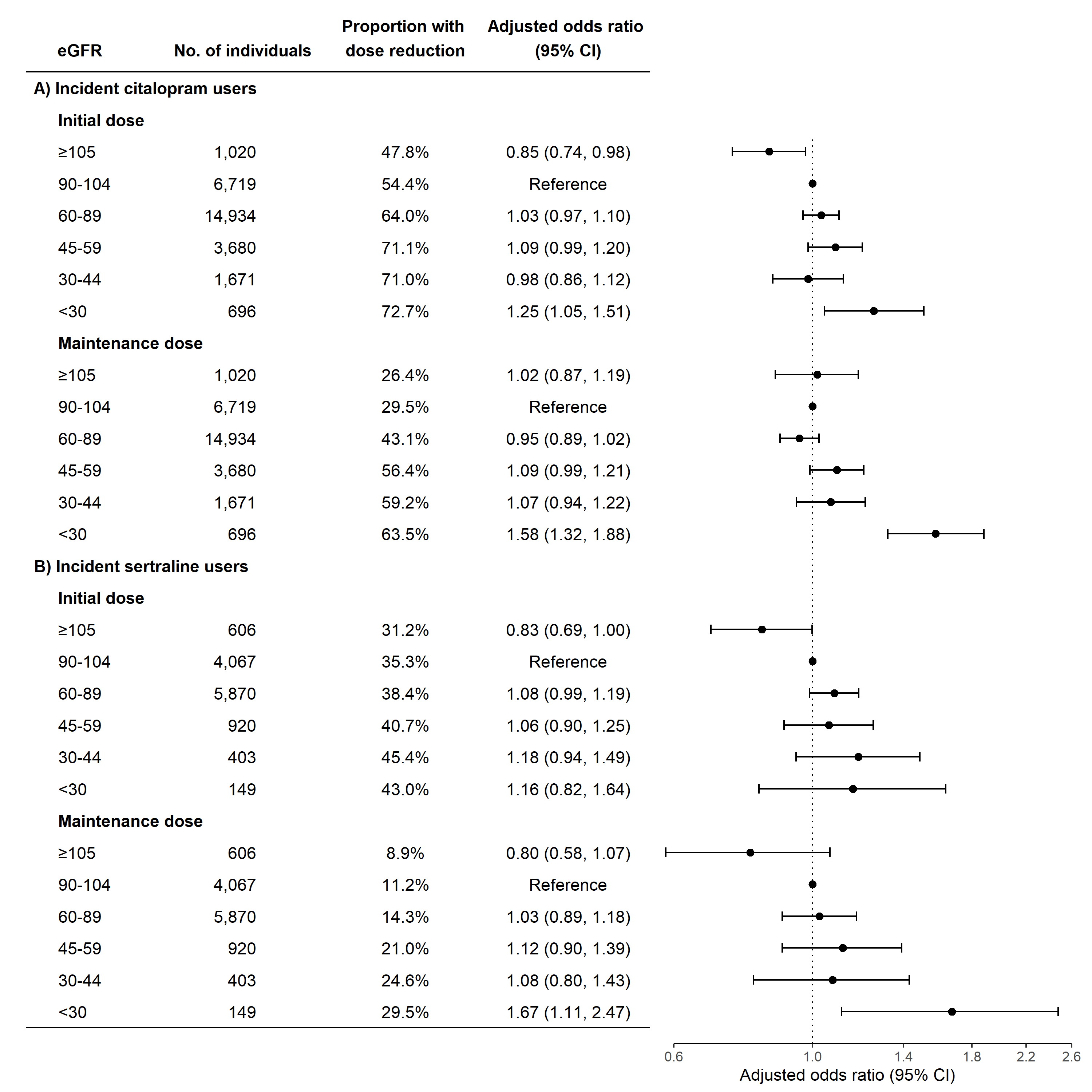


**Supplementary figure 5.** eGFR and dose reductions for citalopram and sertraline

Abbreviations: eGFR, estimated glomerular filtration rate; CI, confidence interval.

Age, sex, psychiatric diagnosis, and use of other psychotropic medications were adjusted for in the model.

**Reference**

1. Pharmaceutical Specialities in Sweden. FASS for Healthcare Staff. The Swedish Association of the Pharmaceutical Industry AB. <https://www.fass.se/LIF/startpage?userType=0>. Published 2021. Accessed 8 May, 2021.

2. Nagler EV, Webster AC, Vanholder R, Zoccali C. Antidepressants for depression in stage 3-5 chronic kidney disease: a systematic review of pharmacokinetics, efficacy and safety with recommendations by European Renal Best Practice (ERBP). *Nephrol Dial Transplant.* 2012;27(10):3736-3745.

3. Hedayati SS, Yalamanchili V, Finkelstein FO. A practical approach to the treatment of depression in patients with chronic kidney disease and end-stage renal disease. *Kidney Int.* 2012;81(3):247-255.

4. The Management of Major Depressive Disorder Working Group. *VA/DoD Clinical Practice Guideline for the Management of Major Depressive Disorder.* Washington, DC: U.S. Government Printing Office; 2022.

5. Ashley C, Dunleavy A. *The renal drug handbook: the ultimate prescribing guide for renal practitioners.* 5th ed. London: CRC Press; 2018.

6. Taylor DM, Barnes TRE, Young AH. *The Maudsley Prescribing Guidelines in Psychiatry.* 13th ed. New Jersey: Wiley Blackwell; 2018.
